# Increased levels of the calcification marker Matrix Gla Protein and the inflammatory markers YKL-40 and CRP in patients with type 2 diabetes and ischemic heart disease

**DOI:** 10.1186/1475-2840-9-86

**Published:** 2010-12-08

**Authors:** Stine B Thomsen, Camilla N Rathcke, Bo Zerahn, Henrik Vestergaard

**Affiliations:** 1 Department of Medicine O, Center of Endocrinology and Metabolism, Copenhagen University Hospital Herlev, Denmark; 2 Department of Clinical Physiology and Nuclear Medicine, Copenhagen University Hospital Herlev, Denmark; 3 Faculty of Health Sciences, University of Copenhagen, Denmark

## Abstract

**Objective and design:**

Low grade inflammation is of pathogenic importance in atherosclerosis and in the development of cardiovascular disease (CVD) and type 2 diabetes (T2D). Matrix GLA protein (MGP), an inhibitor of medial calcification of arteries, is increased in patients with atherosclerosis. In the present study levels of markers of calcification (MGP) and inflammation (YKL-40, hsCRP) were evaluated in patients with T2 D and/or ischemic heart disease (IHD).

**Materials and methods:**

The study population consisted of 1) patients with T2D (n = 45); 2) patients with IHD (n = 37); patients with both T2D and IHD (n = 20) and 4) healthy controls (n = 20). Biochemical parameters were measured in venous blood samples.

**Results:**

Levels of MGP, YKL-40 and hsCRP were increased in patients with IHD and/or T2D (p < 0.0001) and patients with T2D and IHD had higher MGP levels (p < 0.001). In multiple linear regression analyses MGP was associated with patient category (r = 0.36, p < 0.001), and HDL-cholesterol levels (r = 0.29, p < 0.001) adjusting for the significant covariates.

**Conclusions:**

In patients with T2D and/or IHD we found increased levels of plasma MGP indicative of a progressing calcification process. This process is paralleled by increased levels of YKL-40 and hsCRP, which most likely reflect the concomitant low grade inflammatory state in these patients

## Introduction

Low grade inflammation and activation of the innate immune system play a role in the common pathogenesis of both insulin resistance and endothelial dysfunction and subsequently the development of type 2 diabetes (T2D) and atherosclerosis. Several proinflammatory cytokines, acute phase-reactants and cell adhesion molecules seem to play a role in low grade inflammation, and today there is substantial evidence supporting the role of C-reactive protein (CRP), interleukin-6 (IL-6) and tumor necrosis factor-α (TNFα) among others as cardiovascular risk markers and participants in the pathogenesis of obesity, T2D and cardiovascular disease (CVD) [1-4]. CRP still remains the most validated biomarker in this context, although the substantial knowledge about CRP as a predictor of cardiovascular events is being supplemented by studies on emerging markers [5].

Matrix γ-carboxyglutamate (Gla) protein (MGP) is an extracellular matrix protein with wide tissue distribution, not only detectable in the normal blood vessels but also in calcified atherosclerotic plaques [6]. The main function of MGP is to inhibit medial calcification of arteries and thereby to protect the normal environment in the vascular wall [7-9]. Its main sources are cartilage and the vessel wall where it is synthesized by chondrocytes and vascular smooth muscle cells (VSMCs), respectively [8,10,11]. The importance of MGP to prevent calcification in soft tissues *in vivo *has been documented in the MGP knockout mouse model, where calcification of the elastic lamellae in the tunica media resulted in rupture of the large arteries within 8 weeks [8]. The function of MGP as an inhibitor of vascular calcification is exercised in the local tissues and no biological function of circulating MGP has been demonstrated [12]. However, serum MGP levels have been found to be significantly increased in patients with severe atherosclerosis and circulating MGP can be regarded as a marker of atherosclerosis reflecting the degree of inhibition of ongoing calcification processes in the vascular wall. This is consistent with the high MGP mRNA expression observed in atherosclerotic vessels and plaques in patients with type 1 diabetes [9,13].

YKL-40 is a 40 kDa heparin- and chitin-binding glycoprotein which is secreted *in vitro *by a variety of cells. *In vivo *YKL-40 is found in subpopulations of macrophages and VSMCs in different tissues with inflammation and extracellular matrix remodeling as in atherosclerotic plaques [14,15]. YKL-40 induces maturation of monocytes to macrophages and is secreted by macrophages during late stages of differentiation and by activated macrophages [14,15]. YKL-40 has also been shown to be an adhesion and migration factor for vascular cells and it is secernated by differentiated VSMCs [14,15].

The aim of the present study was to evaluate levels of markers of calcification (MGP) and inflammation (YKL-40, hsCRP) in patients with T2D and/or ischemic heart disease (IHD) and in healthy control subjects.

## Materials and methods

### Study population

The study population consisted of 1) patients with T2D (n = 45); 2) patients with IHD (n = 37); patients with both T2D and IHD (n = 20) and 4) healthy glucose tolerant controls (n = 20). Patients with T2D, but without IHD, were all without clinical or biochemical signs of diabetic complications. The patients with T2D were all on oral anti-hyperglycemic treatment, none received insulin. All healthy control subjects were Caucasian and had a sedentary lifestyle, and did not take any medication. Individuals with inflammatory conditions such as ongoing infectious disease/use of antibiotics, rheumatic or connective tissue disorders were excluded from the study. Furthermore, individuals with known chronic obstructive pulmonary disease, kidney disease or cancer were also excluded, because levels of YKL-40 are known to be increased in these conditions [14]. The study was approved by the local ethics committee (H-B-2007-058) and investigations conformed to the principles of The Helsinki Declaration.

### Clinical data

All participants underwent a clinical examination including an ECG. A medical history was obtained and medications were recorded. The following baseline characteristics were also registered: known prior myocardial infarction (MI) and/or previous coronary revascularization procedures (percutaneous coronary intervention and coronary artery bypass grafting). MI was defined according to international guidelines http://www.escardio.org. IHD was defined as a previous episode with increased plasma coronary markers, ECG verified MI or previous coronary revascularization.

All patients with IHD in the study had been referred to a myocardial perfusion imaging if they were considered to have an intermediate risk of having coronary artery disease (CAD) (symptoms of transient chest pain and/or worsening of chest pain when exercising and/or transient referred pain to the upper limbs or neck) or had a history of CAD with renewed suspicion of ischemia. All patients had an abnormal myocardial perfusion. All other patients in the study were without symptoms of cardiovascular disease and had a normal resting ECG.

### Measurements

Analyses of the following markers of inflammations were performed: 1) Human MGP (mAb^3-15^) was measured using an ELISA (Biomedica Medizinprodukte, Vienna, Austria). Standard range was 0-90 nmol/l, lower detection limit was 0.3 nmol/l and intra- and interassay coefficients of variation were 5.5% and 8.0%, respectively. 2) Plasma YKL-40 was measured using an ELISA method (Quidel, USA). Measuring range was 20-300 ng/ml, with intra- and interassay coefficients of variation of 5.8% and 6.0%, respectively. 3) CRP was measured with a highly sensitive, latex-particle-enhanced immunoturbidimetric assay (DAKO, Glostrup, Denmark) with a measuring range of 0.2-80 mg/l and a lower detection limit of 0.03 mg/l.

### Statistical analyses

Following a test of statistical (log-) normality, data are presented as mean ± SD or as median and interquartile range (IQR). Comparisons between the groups were performed by a One-Way ANOVA. A non-parametric test was used if a variable exhibited a clear non-Gaussian distribution. The χ2-test was used for categorical variables. Analyses of associations were performed in the total study population using linear regression models with MGP, YKL-40 or hsCRP as dependent variable. Univariate analyses of correlations of either one of the biomarkers YKL-40, hsCRP and MGP were performed prior to multivariate analyses. Multivariate analyses including age, gender, patient category, cholesterol levels, blood pressure, and presence of IHD or diabetes were performed for biomarkers with significant outcome in the univariate analyses. All p-values were calculated as two-sided, and a p-value < 0.05 was considered significant.

Analyses were made with the statistical software package SPSS15.0 (SPSS inc., Chicago, Il, USA).

## Results

### Clinical data

Clinical characteristics of the different patient categories of the study population are presented in Table 1. There was an equal distribution between genders. Patients with T2D without IHD and the control subjects were younger that patients with IHD (p < 0.001). There was a significant difference in systolic but not in diastolic blood pressure between groups and more patients with both T2D and IHD had hypertension. Significantly lower levels of LDL-cholesterol and higher levels of HDL-cholesterol were found in patients with IHD when compared to patients without IHD (p < 0.0001).

**Table 1:** Clinical characteristics of the study population.

	Control subjects	Patients with T2D	Patients with IHD	Patients with T2D and IHD	P-value Between groups
N	20	45	37	20	
Male (%)	6 (30)	22 (49)	9 (24)	9 (45)	0.1
Age (yrs); range	50 (34-66)	54 (41-73)	66 (47-81)	63 (47-79)	<0.001
Weight (kg)	81,5 ± 14,1	85,1 ± 12,2	82,9 ± 14,6	84,5 ± 15,5	0.57
Body mass index (kg/m^2^)	26,3 ± 3,6	29,5 ± 3,8	28,2 ± 5,5	29,7 ± 5,2	0.02
Duration diabetes (yrs)*	-	2 (0-15)	-	8 (3-26)	<0.001
Diastolic BP (mmHg)	81 ± 8	84 ± 11	87 ± 13	83 ± 10	0.34
Systolic BP (mm Hg)	131 ± 11	141 ± 19	145 ± 25	150 ± 23	0.045
Hypertension (%)	0	14 (31)	9 (24)	13 (65)	<0.001
MI (%)	-	-	26 (70)	8 (40)	
PCI/CABG (%)	-	-	27 (73)	11 (55)	
Total cholesterol (mM)	5,3 ± 1,0	5,7 ± 1,3	4,9 ± 1,3	5,0 ± 1,2	0.026
LDL-cholesterol (mM)	3,8 ± 0,9	4,0 ± 1,3	2,7 ± 1,1	2,5 ± 0,8	<0.001
HDL-cholesterol (mM)	0,89 ± 0,30	0,80 ± 0,41	1,34 ± 0,48	1,27 ± 0,26	<0.001
Creatinine (μM)	NA	82 ± 21	90 ± 22	94 ± 31	0.23
					
ACE (%)	-	13 (27)	18 (49)	15 (71)	<0.001
BB (%)	-	7 (15)	29 (78)	12 (57)	<0.001
CCB (%)	-	5 (10)	7 (19)	5 (24)	0. 1
Statins (%)	-	21 (43)	31 (84)	18 (86)	0.004
Diuretics (%)	-	13 (27)	15 (41)	11 (52)	0.002
NTG (%)	-	-	13 (35)	5 (24)	<0.001

*Median (range); Mean ± SD; Number (%); NA Not analysed

Patients with IHD were more frequently treated with ACE inhibitors, beta blockers (BB), statins and diuretics as compared to T2D patients without IHD (p < 0.001 for ACE, BB and statins; p = 0.002 for diuretics).

### Markers of calcification and inflammation

#### MGP

**Figure 1:**
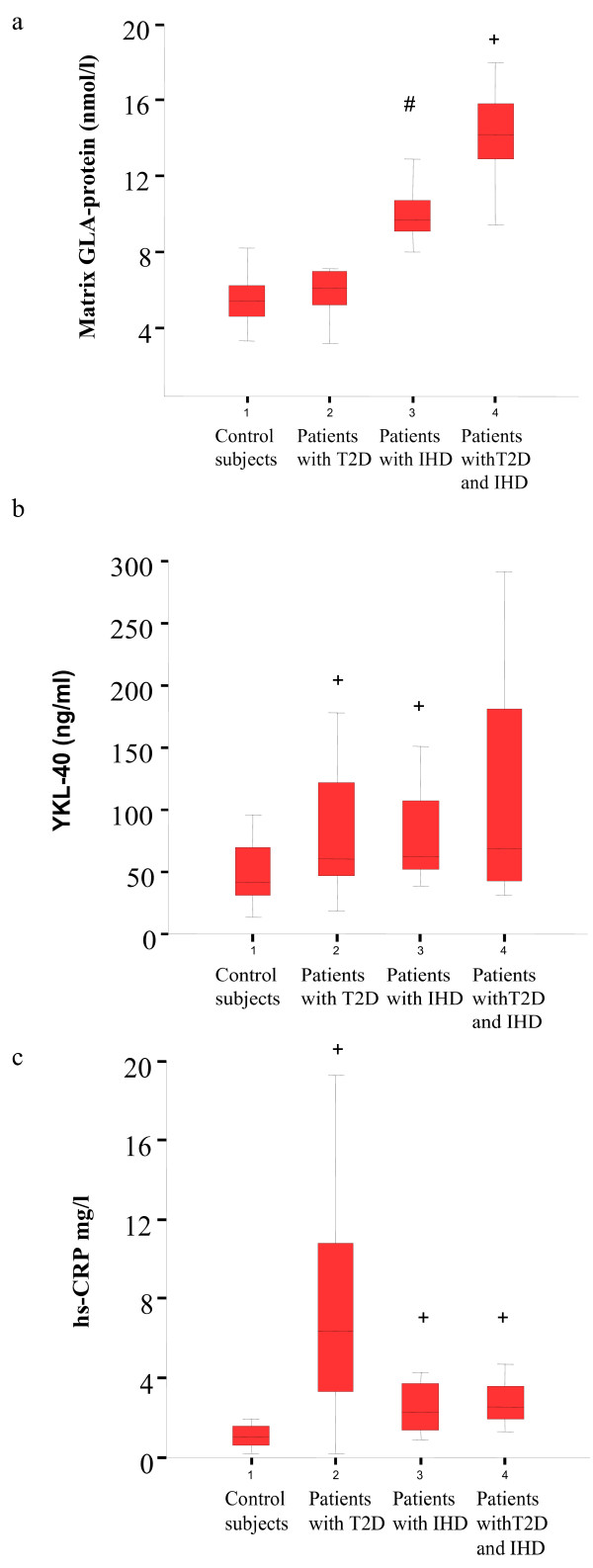
Levels of MGP, YKL-40 and hsCRP were significantly different between the groups (ANOVA, *0.0001 < p < 0,01 for all comparisons) (-T2D/-IHD N = 20; +T2D/-IHD N = 45; -T2D/+IHD N = 37; +T2D/+IHD N = 20). MGP levels were significantly higher in patients with both T2D and IHD when compared to the other groups (+p < 0.001 for all comparisons), and in IHD patients when compared to patients with T2D and controls (#p ≤ 0.007). Levels of YKL-40 were significantly higher in patients with T2D and in patients with IHD when compared to the control subjects (p < 0.03). Levels of hsCRP were significantly higher in patients with T2D with and without IHD when compared to the control group (+p ≤ 0.001 for all).

MGP levels were significantly higher in patients with both T2D and IHD, when compared to patients with either IHD or T2D (p < 0.001 for all comparisons)(Table 2). Moreover, MGP levels in non-diabetic patients with IHD were higher than in T2D patients without IHD (p = 0.007) (Figure 1a). Univariate analyses revealed significant correlations between MGP and age (r = 0.43), HDL- and LDL-cholesterol levels (r = 0.59 and r = -0.47, respectively), LDL:HDL-cholesterol ratio (r = -0.62), statin (r = 0.55) and ACE treatment (r = 0.47) and patient category (r = 0.73) (all p ≤ 0.001) (Table 3). A similar pattern was found in subgroup analyses of patients with either IHD only or T2D only. No correlation was found between MGP levels and serum creatinine levels (r = -0.27, p = 0.45). In the total group of control subjects and IHD patients without T2D, MGP correlated positively with hsCRP (r = 0.50, p < 0.0001) and YKL-40 (r = 0.30; p = 0.03), whereas it correlated negatively to hsCRP in the total group of T2D patients without and with IHD (r = -0.28, p = 0.03). In multiple linear regression analyses MGP was associated with patient category (r = 0.36, p < 0.001), and HDL-cholesterol levels (r = 0.29, p < 0.001) adjusting for the significant covariates.

**Table 2:** Biomarkers

	Control subjects	Patients with T2D	Patients with IHD	Patients with T2D and IHD	P-value Between groups
MGP (nmol/l)	5,4 (4,5-6,3)	6,1 (5,2-7,1)	9,7 (9,1-10,7)	14,2 (12,7-15,9)	<0.001
YKL-40 (ng/ml)	42 (31-72)	61 (47-133)	63 (51-110)	69 (43-184)	= 0.02
hsCRP (mg/l)	1,1 (0,5-1,7)	6,3 (3,2-10,9)	2,3 (1,3-3,9)	2,6 (1,9-3,6)	<0.001

Median (IQR)

**Table 3:** Intercorrelations of MGP.

	Correlation coefficient, r	p value
**Age**	0.43	<0.001
**Patient category**	0.73	<0.001
**LDL-cholesterol**	-0.47	<0.001
**HDL-cholesterol**	0.59	<0.001
**LDL:HDL ratio**	-0.62	<0.001
**Statin treatment**	0.55	<0.001
**ACE treatment**	0.47	<0.001

Log-transformed data, univariate analyses.

Levels of MGP were significantly higher in patients treated with statins (2.32 ± 0.37 vs. 1.83 ± 0.41, p < 0.001) and ACE-inhibitors (2.36 ± 0.43 vs. 1.95 ± 0.43, p < 0.001) compared to non-treated.

#### YKL-40

Levels of YKL-40 were significantly higher in patients with IHD and T2D (p < 0.05) (Figure 1b)). However, levels of YKL-40 did not differ between patients with both T2D and IHD and the control group (p = 0.1). No differences were seen in levels of YKL-40 between patient treated with statins (p = 0.13) and ACE-inhibitors (p = 0.20) (data not shown) when compared to non-treated patients.

Only patient category (r = 0.22, p = 0.04) and LDL: HDL-cholesterol ratio (r = 0.31, p = 0.02) was significantly associated with levels of YKL-40 in a multiple regression model adjusting for the significant covariates.

#### CRP

Levels of hsCRP were significantly higher in any patient group when compared to the control group (p ≤ 0.001 for all comparisons) (Figure 1c). Patients with T2D but no IHD had significantly higher levels of hsCRP compared to patients with IHD (p = 0.008), but not compared to patients with T2D and IHD (p = 0.16). No differences were seen in levels of hsCRP between patient treated with statins (p = 0.14) and ACE-inhibitors (p = 0.86) (data not shown) when compared to non-treated patients.

In a multiple regression analysis hsCRP was only associated with BMI (r = 0.42, p < 0.001) after adjusting for the significant covariates.

None of the biomarker levels differed between genders (p > 0.4).

## Discussion

The present study is to our knowledge the first to compare MGP expression in patients with either T2D or IHD or both. We describe increased serum MGP levels in patients with either T2D or IHD as well as in patients with both T2D and IHD, when compared to healthy control subjects. Significantly higher levels of MGP were found in patients with IHD when compared to patients without IHD, while no difference was seen when comparing patients with and without T2D. A stepwise increase was seen in serum MGP, with the highest levels in patients with both T2D and IHD. Based on our results it seems as if increased MGP not only is a marker of IHD characterized by intima calcification and subsequent atherosclerosis, but also of media sclerosis as typical seen in diabetes since an apparent additive concentration of MGP are documented in patients with both T2D and IHD. As in a previous study, we also found associations of MGP concentrations with levels of HDL- and LDL- cholesterol, consistent with the evidence that traditional lipid risk factors are significantly associated with circulating MGP [13].

Atherosclerosis and vascular calcification are extremely complex mechanism, with a long list of potential derangements on different pathways, and biomarkers have been extensively evaluated and become an important tool, helping to improve patient care [16]. Novel biomarkers of vascular inflammation, atherosclerosis and calcification and advanced glycation have been associated with both macrovascular late complications in high-risk T2D patients and with the severity of atherosclerosis in patients with carotid artery disease and lower limp artery disease [16-20].

In individuals without compromised kidney function as in the present study, levels of circulating MGP depend primarily on the synthesis and secretion and clearance of MGP from VSMC and subsequent binding of MGP to calcified areas within the vascular wall. We found significantly increased levels of serum MGP in patients with T2D and in patients with IHD, when compared to healthy control subjects, which is in agreement with previous studies demonstrating increased serum MGP levels in patients with severe atherosclerosis and in patients with type 1 diabetes [13]. We found an additive effect of T2D and IHD on MGP levels, which has never been demonstrated previously. This effect is probably due to a more advanced and general cardiovascular disease in patients with both T2D and IHD, when compared to patients with either T2D or IHD. Furthermore, MGP levels were significantly higher in non-diabetic patients with IHD compared to patients with T2D without clinical IHD, implying that the calcification process seems more active in conditions with clinical atherosclerosis, but obviously also active in conditions with increased risk of media and intima calcification as in T2D when compared to healthy subjects.

It is also well known that the risk of myocardial infarction (MI) is similar in T2D patients without prior MI and in non-diabetic patients with prior MI, and that the risk is even higher in diabetic patients with previous MI [21]. Vascular calcification occurs at two anatomic sites, in the intima where it is associated with atherosclerosis and in the tunica media as Mönckeberg's sclerosis [22]. Media sclerosis is most commonly seen in patients with diabetes where it develops independent of atherosclerosis, implying different etiological mechanisms. Media calcifications are associated with VSMCs, whereas intima calcification in atherosclerosis occurs in macrophage and lipid rich atherosclerotic lesions. VSMCs grown in culture contain high levels of MGP, which is thought to limit the rate of calcification. MGP is a strong local inhibitor of vascular calcification, and although circulating MGP has no known biological function [12], it may reflect inhibition of calcification processes in the vascular wall. In healthy vessels MGP is synthesized at a low rate, probably because the need for calcification inhibition is low [9,23]. In vessels from patients with diabetes, MGP levels are lower than in normal vessels, which suggest that reduced MGP in diabetes may predispose to calcification [24]. However, levels of MGP has been demonstrated to be high in calcified vessels, where MGP most probably is expressed as an inhibitory counteraction of the calcification process [6,23].

Inflammation is probably an initial event very early on in plaque formation leading to calcification and IHD [25]. It has been demonstrated that statins, through their capacity to inhibit inflammation, can reduce the increased osteogenic and inflammatory activities seen with plaque progression, and this is correlated with the reduction in calcification [26]. In the present study we found that significantly more patients with IHD were treated with statins, so we cannot exclude that this has influenced the levels of MGP. However, since statin treatment reduces inflammation and can ameliorate the calcification response in the vessel wall, higher MGP levels would be expected had the patients been without statin treatment.

Taken together, our and previous studies suggest that arterial calcification may lead to increased MGP expression, probably in a feedback attempt to reduce bone-like formation of calcium deposits in the vessel walls. However, it is still unclear whether serum MGP levels reflect activation, tissue production and deposition.

Several studies have documented, that low-grade inflammation and activation of the innate immune system is involved in the common pathogenesis to atherosclerosis, endothelial dysfunction, insulin resistance and the development of T2D. We found significantly increased YKL-40 concentrations in patients with either T2D and/or IHD, thus confirming previous studies [27-30]. We also found a correlation between YKL-40 and patient category. As in a previous study of patients with T2D [27,28], no correlation was found between hsCRP and YKL-40 implying, that YKL-40 and CRP are produced and secernated independently of each other.

Studies have shown, that increased levels of YKL-40 are independently associated with the presence and extent of CAD [29-32]. In patients with myocardial infarction even higher YKL-levels are documented [31]. YKL-40 has also been found to be associated with all-cause as well as cardiovascular mortality in both patients with stable IHD [31] and in the general population above 50 years of age without known diabetes or IHD [28]. In patients with type 1 diabetes, increasing levels of YKL-40 are seen with increasing levels of albuminuria, suggesting that YKL-40 might be able to be used as an early marker of CVD [33]. The present finding seems to support this hypothesis.

It is well-known that serum CRP is increased in T2D patients [1]. We found a 7-fold higher concentration of serum hsCRP among T2D patients without IHD and a 3-fold higher in T2D patients with IHD, when compared to healthy control subjects. Furthermore, we found a strong correlation between serum hsCRP and BMI and patient category. It is also well known that CRP is a cardiovascular marker even within ranges considered normal [34-36]. In the present study we found a nearly 4-fold higher concentration of serum hsCRP in non-diabetic patients with IHD together with a strong correlation between serum hsCRP and BMI and HDL-cholesterol levels, in patients with and without IHD. Levels of hsCRP were significantly lower in non-diabetic patients with IHD compared to patients with T2D without IHD. Based on our and previous studies it can be speculated, that the low grade inflammation seen in both IHD and T2D declines in diabetes, when diabetic patients develop symptomatic atherosclerosis, and therefore make hsCRP as a biomarker of IHD in diabetic patients questionable.

In the present study, more patients with IHD received statin treatment. Statins inhibit inflammation, and early treatment of inflammation seems to ameliorate the calcification response in the vessel wall [26]. We found increased levels of the inflammatory markers YKL-40 and hsCRP in patients with T2D and IHD, when compared to control subjects, however no differences were found in levels of these inflammatory markers between statin treated and non-treated patients. Statin treatment in especially IHD patients could also influence the levels of both YKL-40 and hsCRP, but without this treatment we would expect even higher levels of both markers in the IHD groups. In a recent study of patients with stable coronary artery disease (CAD), it was shown that YKL-40 levels were significantly reduced in statin treated patients compared to non-treated patients [37]. The median YKL-40 levels were 77% higher in the non-treated CAD group compared to the non-treated patients in our study, and median YKL-40 levels in statin treated CAD group were comparable to our statin treated patients, so we do not think that the participants in the two studies are comparable.

We found that patients treated with statins had higher levels of MGP, and since we also found a positive correlation between HDL levels and MGP and a negative correlation between LDL levels and MGP, it could be speculated that higher MGP levels were an indication of better clinical and prognostic regulation of lipid levels due to statin treatment. Higher MGP levels could therefore be a positive indicator for the prognosis due to its function as an inhibitor of medial calcification of arteries. In a multiple regression analyses there were however no association between MGP levels and statin use or LDL-levels, adjusting for significant covariates, but we do not now if MGP can be used as a biomarker or positive indicator for prognosis of IHD.

In conclusion, in patients with T2D and IHD we found increased levels of plasma MGP which may indicate progressing media and intima calcification processes. Moreover, these processes were paralleled by increased inflammation, which may reflect an initial event very early on in the processes leading to calcification

## Conflicts of interest

The authors declare that they have no financial or non-financial competing interests.

## Authors' contributions

SBT participated in the design, coordination of the study, and interpretation of data and drafted the manuscript. CNR participated in analysis and interpretation of data, and helped to draft the manuscript. BZ participated in acquisition of data and conducts of the study and helped to draft the manuscript. HV conceived of the study and participated in the design, coordination, conduct of the study, performed the statistical analysis and helped to draft the manuscript. All authors have read and approved the final version of the manuscript.
